# Comment on: Once after a full moon: acute type A aortic dissection and lunar phases

**DOI:** 10.1093/icvts/ivad049

**Published:** 2023-03-23

**Authors:** Fabian Junker, Tristan Ehrlich, Hans-Joachim Schäfers

**Affiliations:** Department of Thoracic and Cardiovascular Surgery, Saarland University Medical Center, Homburg/Saar, Germany; Department of Thoracic and Cardiovascular Surgery, Saarland University Medical Center, Homburg/Saar, Germany; Department of Thoracic and Cardiovascular Surgery, Saarland University Medical Center, Homburg/Saar, Germany

**Keywords:** Aortic dissection, Lunar phases, Seasons

Dear Editor,

We read with interest the recent publication on patterns in the occurrence of aortic dissection [1]. Lunar phases have been related to a number of aspects, such as tides, or weather phenomena. We feel that the relationship between lunar phases and the occurrence of acute aortic dissection type A requires a closer look.

In interpreting the results, one has to consider the geographic characteristics of the region studied, since findings may not be transferable to other regions.

Scandinavia—particularly in its Northern parts—is characterized by marked variations of day/night rhythm depending on season, which may have secondary effects. There is already evidence that the different seasons may have an influence on the prevalence of cardiovascular diseases in general [2]. The long days in summer may lead to sleep deprivation, which in turn is a risk factor for hypertension [3, 4], which is a known risk factor for dissection [5, 6]. Long nights in winter have been associated with a higher prevalence of depression [7]; under such circumstances an influence of moon light or its vanishing phase appears conceivable [4].

Based on the publication [1], we are uncertain whether the data can be generalized or whether this phenomenon is in fact restricted, for example, to the Northern regions. One also needs to keep in mind that populations may express different distributions of risk factors. For example, the incidence of acute aortic dissection type A in an all-white US cohort is about 4.4 per 100 000 patients [6], whereas in Scandinavia, it is about 8.7 per 100 000 patients [5].

Possibly a closer look at more detailed information is necessary, such as season or more precise location, e.g. Northern regions with pronounced extremes of day/night rhythm. This could shed more light on the variations in disease expression and may contribute to the improvement of prevention and therapy.
